# A chromosomally encoded T7 RNA polymerase-dependent gene expression system for *Corynebacterium glutamicum*: construction and comparative evaluation at the single-cell level

**DOI:** 10.1111/1751-7915.12236

**Published:** 2014-12-09

**Authors:** Maike Kortmann, Vanessa Kuhl, Simon Klaffl, Michael Bott

**Affiliations:** Institute of Bio- and Geosciences, IBG-1: Biotechnology, Forschungszentrum JülichJülich, D-52425, Germany

## Abstract

*Corynebacterium glutamicum* has become a favourite model organism in white biotechnology. Nevertheless, only few systems for the regulatable (over)expression of homologous and heterologous genes are currently available, all of which are based on the endogenous RNA polymerase. In this study, we developed an isopropyl-β-d-1-thiogalactopyranosid (IPTG)-inducible T7 expression system in the prophage-free strain *C. glutamicum* MB001. For this purpose, part of the DE3 region of *Escherichia coli* BL21(DE3) including the T7 RNA polymerase gene *1* under control of the *lac*UV5 promoter was integrated into the chromosome, resulting in strain MB001(DE3). Furthermore, the expression vector pMKEx2 was constructed allowing cloning of target genes under the control of the T7*lac* promoter. The properties of the system were evaluated using *eyfp* as heterologous target gene. Without induction, the system was tightly repressed, resulting in a very low specific eYFP fluorescence (= fluorescence per cell density). After maximal induction with IPTG, the specific fluorescence increased 450-fold compared with the uninduced state and was about 3.5 times higher than in control strains expressing *eyfp* under control of the IPTG-induced *tac* promoter with the endogenous RNA polymerase. Flow cytometry revealed that T7-based *eyfp* expression resulted in a highly uniform population, with 99% of all cells showing high fluorescence. Besides *eyfp*, the functionality of the corynebacterial T7 expression system was also successfully demonstrated by overexpression of the *C. glutamicum pyk* gene for pyruvate kinase, which led to an increase of the specific activity from 2.6 to 135 U mg^−1^. It thus presents an efficient new tool for protein overproduction, metabolic engineering and synthetic biology approaches with *C. glutamicum*.

## Introduction

The recombinant production of proteins is a highly important issue in industrial biotechnology as well as in scientific research. Many different expression systems have been established in various eukaryotic and prokaryotic organisms (Demain and Vaishnav, 2009). Due to their easy handling and well-established genetic tools, bacteria are broadly used to express heterologous and homologous genes (Baneyx, 1999; Terpe, 2006; Chen, 2012). One of the most popular and commonly used systems for high-level protein production in *Escherichia coli* is the T7 expression system developed by Studier and Moffatt (1986). It is based on the RNA polymerase (RNAP) of bacteriophage T7, which shows a number of beneficial properties: (i) single-subunit enzyme in contrast to multi-subunit bacterial RNAP, (ii) high processivity, (iii) high specificity towards the T7 promoter, (iv) independence of auxiliary transcription factors, (v) production of very long transcripts, and (vi) termination only by class I and class II termination signals that differ significantly from bacterial transcription termination sites (Chamberlin and Ring, 1973; Macdonald *et al*., 1994; Lyakhov *et al*., 1998). Expression hosts like *E. coli* BL21(DE3) carry a single copy of gene *1* for T7 RNAP located chromosomally on a λDE3 lysogen (Studier and Moffatt, 1986). In strain *E. coli* BL21(DE3), transcription of gene *1* is controlled by a *lac*UV5 promoter, allowing repression by LacI and induction with isopropyl-β-d-1-thiogalactopyranoside (IPTG). The expression of desired target genes is controlled by the T7 promoter, which is usually present on a suitable expression vector. To minimize basal transcription, a LacI binding site can be introduced in front of the target gene, placing both gene *1* and the target gene under the control of the LacI repressor (Dubendorff and Studier, 1991). The characteristics of the T7 RNAP-dependent expression system permit a very efficient and exclusive expression of genes under control of the strong T7 promoter. Due to its favourable properties, the T7 RNAP-based expression system has also been established in a variety of other bacteria, such as *Pseudomonas aeruginosa* (Brunschwig and Darzins, 1992), *Pseudomonas putida* (Herrero *et al*., 1993), *Ralstonia eutropha* (Barnard *et al*., 2004), *Bacillus megaterium* (Gamer *et al*., 2009), *Streptomyces lividans* (Lussier *et al*., 2010), *Rhodobacter capsulatus* (Katzke *et al*., 2010; Arvani *et al*., 2012) and *Corynebacterium acetoacidophilum* (Equbal *et al*., 2013).

*Corynebacterium glutamicum* is a Gram-positive soil bacterium of the order *Corynebacteriales* and serves in industry as the major host for production of amino acids, with l-glutamate and l-lysine being the most important ones. Efficient strains are available also for the synthesis of a variety of other amino acids, for example l-leucine (Vogt *et al*., 2013), l-serine (Stolz *et al*., 2007) or d-serine (Stäbler *et al*., 2011). Furthermore, a variety of other commercially interesting metabolites can be produced with *C. glutamicum* (Becker and Wittmann, 2012), such as organic acids (Wendisch *et al*., 2006; Okino *et al*., 2008; Litsanov *et al*., 2012a,b; Wieschalka *et al*., 2013), diamines (Mimitsuka *et al*., 2007; Kind and Wittmann, 2011; Schneider and Wendisch, 2011) or alcohols (Inui *et al*., 2004; Smith *et al*., 2010; Blombach *et al*., 2011; Yamamoto *et al*., 2013). Despite its complex cell envelope (Bansal-Mutalik and Nikaido, 2011; Marchand *et al*., 2012; Laneelle *et al*., 2013), *C. glutamicum* is also an efficient host for the secretory production of heterologous proteins (see Kikuchi *et al*., 2008; Scheele *et al*., 2013; Matsuda *et al*., 2014; and references therein). Based on the broad spectrum of products and its robustness in large-scale production processes, *C. glutamicum* has become a platform and model organism in industrial biotechnology (Eggeling and Bott, 2005; Burkovski, 2008; Yukawa and Inui, 2013).

The development of production strains often requires the controlled expression of target genes or operons. All currently available systems for controlling gene expression in *C. glutamicum* are based on transcription by the endogenous RNA polymerase (Kirchner and Tauch, 2003; Eggeling and Reyes, 2005; Nesvera and Patek, 2011; Patek *et al*., 2013). In this study, we constructed an IPTG-inducible expression system in *C. glutamicum* that is based on T7 RNAP. We characterized the properties of this system with the *eyfp* gene for enhanced yellow fluorescent protein (Perez-Jimenez *et al*., 2006), which allows for analysing population heterogeneity by flow cytometry, and the homologous *pyk* gene for pyruvate kinase as a test case for overproduction of a cytosolic enzyme. The results obtained show that the T7 system allows very efficient and controllable protein overproduction in *C. glutamicum* to levels that outperform currently available systems.

## Results and discussion

### Construction of a T7 RNAP-dependent expression system for *C. glutamicum*

**Figure 1:**
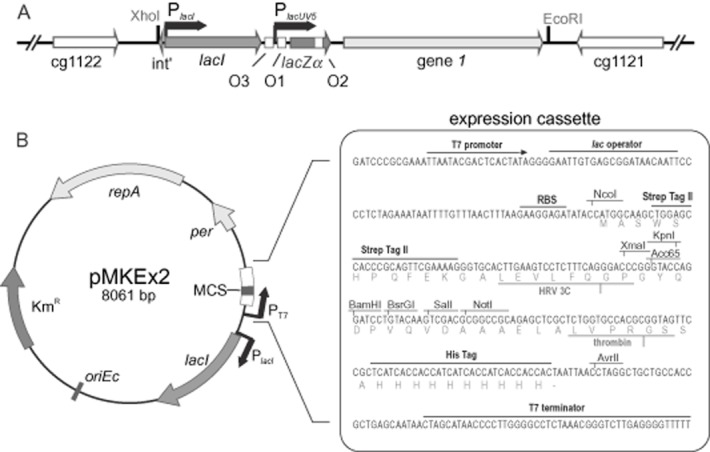
A. genomic region of *C. glutamicum* MB001(DE3) carrying the DE3 insertion. A 4.5 kb DNA fragment was amplified from chromosomal DNA of *E. coli* BL21(DE3) and inserted into the intergenic region of cg1121-cg1122 of MB001(DE3). The fragment contains *lacI*, *lacZ*α and T7 gene *1*, the latter two under the transcriptional control of the *lac*UV5 promoter and its three LacI operator sites O1-O3. B. Map of the expression plasmid pMKEx2, which is based on pJC1 and an expression cassette from pET52b. The region between the T7 promoter and the T7 terminator is shown in detail.

In this study, a T7 RNAP-dependent expression system was developed for *C. glutamicum* based on a chromosomally encoded T7 RNAP and a vector in which the target gene was placed under the control of a T7 promoter. For regulatable chromosomal expression of the T7 RNAP gene *1*, a 4.47 kb fragment (sequence is shown Fig. S1) was amplified by polymerase chain reaction (PCR) from the genome of *E. coli* BL21(DE3) (Table 1) with oligonucleotides DE3-for and DE3-rev (Table 2) that contains the repressor gene *lacI* under the control of its native promoter, *lacZ*α, and T7 gene *1*, the latter two under the control of the *lac*UV5 promoter, including three LacI operator sites O1-O3 (Fig. 1A). The fragment was used to construct plasmid pK18*mobsacB*-DE3 (Table 1), in which the DE3 insert is flanked by adjacent 800 bp regions covering the genes cg1121 (encoding a permease of the major facilitator superfamily) and cg1122 (encoding a putative secreted protein) and their downstream regions (Fig. 1A). Via homologous recombination (Niebisch and Bott, 2001), the DE fragment was integrated into the intergenic region of cg1121-cg1122 within the genome of *C. glutamicum* MB001 (NC_022040.1), a prophage-free derivative of the type strain ATCC 13032, which showed a higher expression level for eYFP than the parent strain (Baumgart *et al*., 2013b). The insertion site is located 340 bp downstream of the cg1121 stop codon. Kanamycin-sensitive and sucrose-resistant clones were checked by PCR and sequence analysis for the correct chromosomal insertion of the DE fragment and the generated strain was named *C. glutamicum* MB001(DE3).

**Table 1:** Bacterial strains and plasmids used in this study

Strain or plasmid	Relevant characteristics	Source
Strain		
*E. coli* BL21(DE3)	F^-^ *ompT hsdSB*(r_B_^-^ m_B_^-^) *gal dcm* (*lcIts857 ind1 Sam7 nin5 lac*UV5*-*T7 gene *1*)	(Studier and Moffatt, 1986)
*E. coli* DH5α	F^-^ φ80*lacZ*ΔM15 Δ(l*acZYA-argF*)U169 *recA*1 *endA*1 *hsdR*17(rk^-^, mk^+^) *phoA supE44 thi*-1 *gyrA*96 *relA*1 λ^-^	Invitrogen
*C. glutamicum* MB001	Type strain ATCC 13032 with deletion of prophages CGP1 (cg1507-cg1524), CGP2 (cg1746-cg1752), and CGP3 (cg1890-cg2071)	(Baumgart *et al*., 2013b)
*C. glutamicum* MB001(DE3)	MB001 derivative with chromosomally encoded T7 gene *1* (cg1122-P*_lacI_-lacI*-P*_lac_*_UV5_ –*lacZ*α-T7 gene *1*-cg1121)	This study
Plasmid		
pEKEx2	Kan^R^; *C. glutamicum*/*E. coli* shuttle vector for regulated gene expression (P*_tac_*, *lacI*^q^, pBL1 *oriV_Cg_*, pUC18 *oriV_Ec_*)	(Eikmanns *et al*., 1991)
pEKEx2-*eyfp*	Kan^R^; expression plasmid carrying the *eyfp* gene under the control of the *tac* promoter	(Hentschel *et al*., 2013)
pET-52b(+)	Amp^R^; *E. coli* vector for expression of target genes under control of the T7 promoter (pBR322 *ori_Ec_*, P_T7_, *lacI*)	Novagen
pJC1	Kan^R^; *E. coli*/*C. glutamicum* shuttle vector (pHM1519 *ori_Cg_*, pACYC177 *ori_Ec_*);	(Cremer *et al*., 1990)
pJC1-*venus*-term	Kan^R^; pJC1 derivative containing *venus* gene and additional terminators	(Baumgart *et al*., 2013a)
pJC1ΔBXS	Kan^R^; pJC1 derivative lacking BamHI, XbaI, and SalI restriction sites	This study
pJC1-P_*tac*_-*eyfp*	Kan^R^; pJC1 derivative containing the *eyfp* gene under the control of the P*_tac_*	This study
pK18*mobsacB*	Kan^R^; vector for allelic exchange in *C. glutamicum* (oriT oriV*_Ec_ sacB lacZ*α)	(Schäfer *et al*., 1994)
pK18*mobsacB*-DE3	Kan^R^; pK18*mobsacB* derivative containing the 4.5 kb λDE3 region (P*_lacI_*, *lacI*, P*_lac_*_UV5_, gene *1*) from *E. coli* BL21(DE3) flanked by two 800-bp DNA regions for homologous recombination into the intergenic region of cg1121 and cg1122	This study
pMKEx2	Kan^R^; *E. coli*/*C. glutamicum* shuttle vector based on pJC1 for expression of target genes under control of the T7 promoter (P*_lacI_*, *lacI*, P_T7_, *lacO1*, N-term. Strep•tag II, MCS, C-term. His•tag, pHM1519 *ori_Cg_*; pACYC177 *ori_Ec_*)	This study
pMKEx2-*eyfp*	Kan^R^; pMKEx2 derivative containing the *eyfp* gene under control of P_T7_	This study
pMKEx2-*pyk*	Kan^R^; pMKEx2 derivative with the *C. glutamicum pyk* gene under control of P_T7_	This study

**Table 2:** Oligonucleotides used in this study

Name	Sequence (5′→3′)	Restriction sites
DE3-for	CCGCTCGAGAACTGCGCAACTCGTGAAAGG	XhoI
DE3-rev	CGGAATTCGTTACGCGAACGCGAAGTC	EcoRI
pETEx-for	AACTGCAGGGAGCTGACTGGGTTGAAGG	PstI
pETEx-rev	AACTGCAGCTTAATGCGCCGCTACAGGG	PstI
pEKEx-for	GGAATTCCATATGTCGCTCAAGCCTTCGTCACTG	NdeI
pEKEx-rev	GGACTAGTTTATCTAGACTTGTACAGCTCGTCCATG	SpeI
eYFP-for	CATGCCATGGACAATAACGATCTCTTTCAGGCATCAC	NcoI
eYFP-rev	CGGGATCCTCAGCCCGCGAGCACC	BamHI
PykCg-for	CGGGATCCGGCGTGGATAGACGAACTAAG	BamHI
PykCg-rev	TGTACAGACACCACGTACAGTGTCAACGC	BsrGI

For exclusive transcription by T7 RNAP, the target gene has to be under control of a T7 promoter. Therefore, a suitable expression vector was constructed based on the shuttle vector pJC1, which contains the pHM1519 replicon for *C. glutamicum* and the pACYC177 replicon for *E. coli* (Cremer *et al*., 1990). In order to provide unique restriction sites for BamHI, XbaI and SalI in the multiple cloning site (MCS) to be inserted, the corresponding restriction sites in the backbone sequence of pJC1 were deleted in advance. For this purpose, pJC1 was digested with BamHI and SalI, the resulting 5′-overhangs filled in with Klenow polymerase and re-circularized via blunt end ligation, resulting in pJC1ΔBXS. A 1.97 kb fragment of plasmid pET52b(+) containing *lacI*, the T7 promoter, the *lac* operator and the downstream MCS was amplified with the oligonucleotides pETEx-for and pETEx-rev and inserted into the unique PstI restriction site of pJC1ΔBXS. After insertion of the expression cassette, an NcoI restriction site in the pJC1 backbone was removed by exchanging a single base (CCATTG → CCATAG). The resulting expression vector was named pMKEx2 (GenBank accession number KM658503) and allows fusion of the target protein either with an n-terminal Streptag II or with a c-terminal decahistidine tag (Fig. 1B).

### Characterization of T7 RNAP-dependent expression system in *C. glutamicum* with the heterologous target protein eYFP and comparison with P_*tac*_-based expression

To analyse the functionality and efficiency of the newly constructed T7 expression system in *C. glutamicum*, the heterologous model protein eYFP (Perez-Jimenez *et al*., 2006) was used as target, as it allows easy detection also at the single-cell level by fluorescence microscopy and fluorescence-activated cell sorting (FACS). The *eyfp* gene was amplified from the plasmid pEKEx2-*eyfp* (Hentschel *et al*., 2013) with oligonucleotides eYFP-for and eYFP-rev and cloned as NcoI-BamHI fragment into pMKEx2 under transcriptional control of the T7 promoter. The resulting plasmid pMKEx2-*eyfp* was transferred into *C. glutamicum* MB001(DE3), and for control purposes into *C. glutamicum* MB001. The synthesis of eYFP by strain MB001(DE3)/pMKEx2-*eyfp* was compared with strain MB001/pEKEx2-*eyfp*. The well-established expression vector pEKEx2 permits expression of target genes under control of the IPTG-inducible *tac* promoter by the endogenous RNA polymerase (Eikmanns *et al*., 1991).

**Figure 2:**
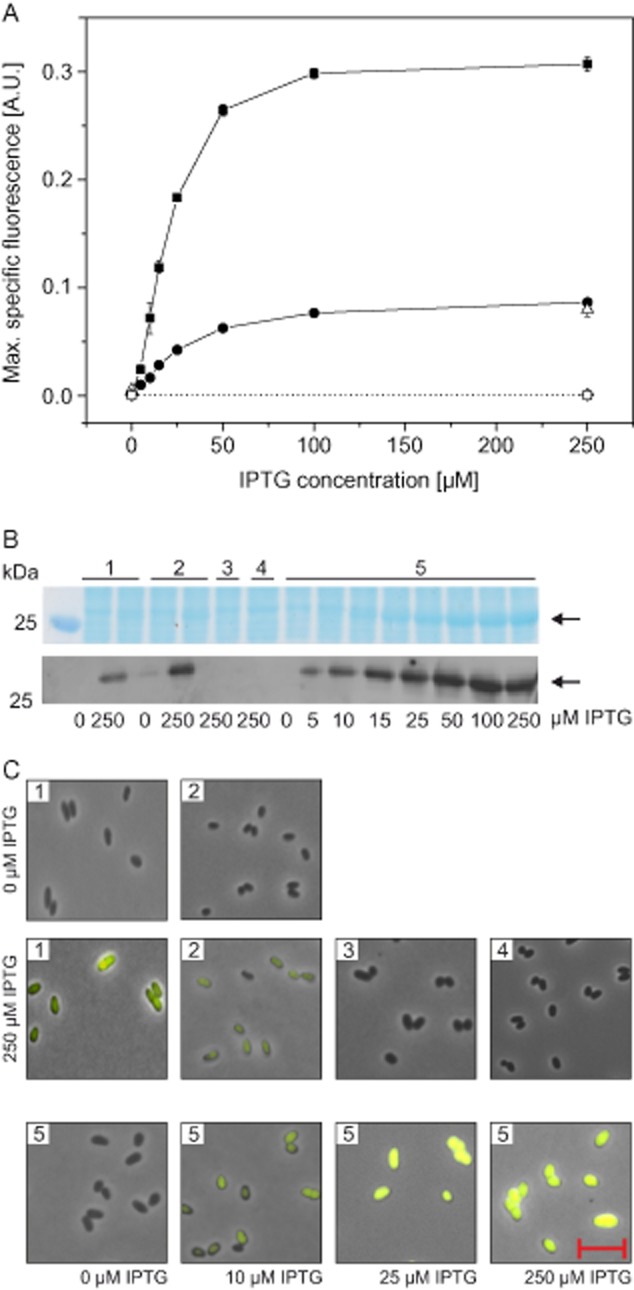
Comparison of eYFP synthesis in *C. glutamicum* with the newly constructed T7-based expression system and the pEKEx2 system using the *tac* promoter and the endogenous RNAP. The strains MB001/pEKEx2-*eyfp* (•/1), MB001/pJC1-P*_tac_*-*eyfp* (Δ/2), MB001(DE3)/pMKEx2 (◊/3), MB001/pMKEx2-*eyfp* (□/4) and MB001(DE3)/pMKEx2-*eyfp* (▪/5) were grown aerobically in CGXII minimal medium with 4% (wt/vol) glucose using a Biolector system at 30°C and 1200 r.p.m. Target gene expression was induced 2 h after inoculation by addition of 0–250 μM IPTG.A. 25 h after starting the cultivation, the maximal specific eYFP fluorescence (ratio of fluorescence emission at 532 nm and backscatter value at 620 nm) was determined. Mean values of at least three independent experiments and standard deviations are shown.B. For protein analysis, cells were disrupted by beat-beating and equivalent amounts of total protein (10 μg) of the cell-free extracts were subjected to SDS-PAGE and visualized by staining with Coomassie Brilliant Blue. In addition, eYFP was detected by Western blotting with an polyclonal anti-GFP antibody. The arrows indicate the predicted size of 27.2 kDa for eYFP.C. Cells were analysed by fluorescence microscopy and images were taken with an exposure time of 40 ms. The red bar represents a length of 5 μm.

The strains were cultivated in CGXII minimal medium with 4% (wt/vol) glucose using a BioLector system (Fig. S2) in the presence of 0, 5, 10, 15, 25, 50, 100 and 250 μM IPTG and expression of the target gene was determined by measuring the specific eYFP fluorescence (Fig. 2). For strain MB001/pMKEx2-*eyfp*, which lacks T7 RNAP, only background specific fluorescence was observed (< 0.001). In the case of strain MB001(DE3)/pMKEx2-*eyfp*, specific fluorescence was very low in the absence of IPTG (< 0.001), but increased up 0.30 when the medium was supplemented with 100 μM IPTG. These results demonstrate that the T7 promoter is not recognized by the corynebacterial RNAP, whereas specific and efficient expression occurs in the presence of T7 RNAP. In the case of strain MB001/pEKEx2-*eyfp*, the specific fluorescence in the absence of IPTG (0.001) was 1.6-fold higher than in the case of MB001(DE3)/pMKEx2-*eyfp* and increased up to 0.08 in the presence of 100 μM IPTG (Fig. 2). Thus, the T7 system shows a slightly lower expression level in the absence of IPTG and an up to fourfold higher maximal expression level in the presence of IPTG compared with pEKEx2-based expression of *eyfp*. Half-maximal specific fluorescence was obtained with 20 μM IPTG for strain MB001(DE3)/pMKEx2-*eyfp* and with 31 μM IPTG for strain MB001(DE3)/pEKEx2-*eypf*.

To exclude an influence of the different replication mechanisms of pMKEx2 and pEKEx2, the specific eYFP fluorescence was also determined in *C. glutamicum* MB001(DE3) transformed with plasmid pJC1-P*_tac_*-*eyfp*. This plasmid contains the same replicon as pMKEx2 and the expression cassette of pEKEx2 (for construction see *Experimental procedures*). The results obtained with MB001/pJC1-P*_tac_*-*eyfp* were comparable to that of MB001/pEKEx2-*eyfp*, indicating that the difference to T7-based *eyfp* expression is not caused by the different replicons, but by the use of different RNAPs and promoters (Fig. 2).

To further characterize the T7 expression system in *C. glutamicum*, the four strains described above were analysed by SDS-PAGE, Western blotting and fluorescence microscopy. The Coomassie-stained SDS-polyacrylamide gel and more clearly the Western blot with anti-green-fluorescent protein (GFP) antiserum shown in Fig. 2B qualitatively confirmed the results of the specific fluorescence measurements described above. In the case of strains MB001/pEKEx2-*eyfp* and MB001(DE3)/pMKEx2-*eyfp*, no distinct band with a size of 27 kDa (calculated mass of eYFP) was visible in cells grown in the absence of IPTG, whereas a faint band was visible in the case of strain MB001/pJC1-P*_tac_*-*eyfp*. When cultivated in the presence of 250 μM IPTG, the 27 kDa eYFP protein band was clearly visible in strains MB001/pEKEx2-*eyfp*, MB001/pJC1-P*_tac_*-*eyfp* and pMB001(DE3)/pMKEx2-*eyfp*. For the latter strain, the intensity of the eYFP band continuously increased when the IPTG concentration was raised from 5 μM to 100 μM. The eYFP bands of MB001/pEKEx2-*eyfp* and MB001/pJC1-P*_tac_*-*eyfp* in the presence of 250 μM IPTG were much fainter than those of strain MB001(DE3)/pMKEx2-*eyfp* in the presence of 50, 100 and 250 μM IPTG. As expected, no eYFP band was visible in the negative control strains MB001(DE3)/pMKEx2 and MB001/pMKEx2-*eyfp* in the presence of 250 μM IPTG. The fluorescence microscopy images shown in Fig. 2C were also in agreement with the results of the specific fluorescence measurements, the SDS polyacrylamide gels and the Western blots, with the most strongly fluorescent cells being those of strain MB001(DE3)/pMKEx2-*eyfp* cultivated in the presence of 250 μM IPTG.

### Characterization of T7 RNAP-dependent expression system in *C. glutamicum* with the heterologous target protein eYFP and comparison with P_*tac*_-based expression at the single-cell level

**Figure 3:**
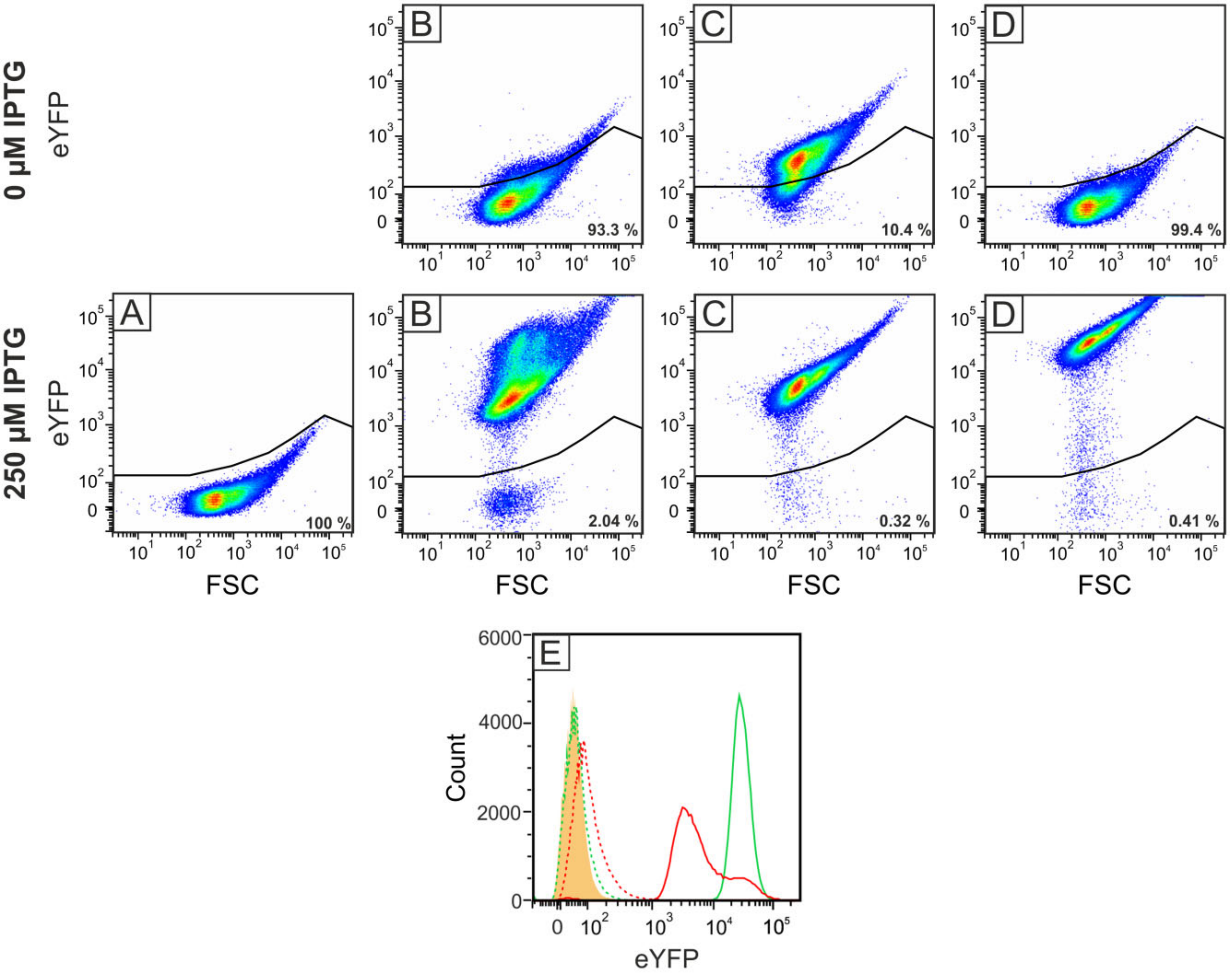
Analysis of heterologous eYFP production in the *C. glutamicum* strains MB001/pMKEx2-*eyfp* (A), MB001/pEKEx2-*eyfp* (B), MB001/pJC1-P*_tac_*-*eyfp* (C) and MB001(DE3)/pMKEx2-*eyfp* (D) at the single-cell level. The strains were cultivated for 24 h at 30°C in CGXII minimal medium with 4% (wt/vol) glucose using a Biolector system. Induction of *eyfp* expression was triggered by adding 250 μM IPTG to the cultures after 2 h. Pseudo-coloured dot plots from flow cytometry analysis (excitation at 488 nm, emission at 533 nm) of at least 100 000 cells of each strain displaying the eYFP fluorescence signal against the forward scatter signal (FSC) are shown. The gate used to define non-fluorescent cells was set with *C. glutamicum* MB001(DE3)/pMKEx2 with 100% of the cells falling into this gate (data not shown). The number inside the blot indicates the percentage of cells inside this gate. Panel E shows a histogram of the strains MB001/pEKEx2-*eyfp* (red) and MB001(DE3)/pMKEx2-*eyfp* (green). The number of cells is plotted against the eYFP fluorescence intensity. The dotted peaks show the measurement of the uninduced culture, the continuous line the cultures grown in the presence of 250 μM IPTG. The orange peak represents the background set with strain MB001(DE3)/pMKEx2.

Flow cytometry was used to analyse *eyfp* expression at the single-cell level, allowing the detection of population heterogeneity (Figs 3 and S3). The gate used to define background fluorescence was set with *C. glutamicum* MB001(DE3)/pMKEx2, with 100% of the analysed cells falling into this gate (Fig. 3A). In the case of strain MB001/pEKEx2-*eyfp* (Fig. 3B), 7% of the cells cultivated in the absence of IPTG showed fluorescence above background, with an average intensity of 1.0 × 10^2^, confirming the leakiness of the *tac* promoter used. When cultivated in the presence of 5, 10, 15, 25, 50, 100 and 250 μM IPTG, MB001/pEKEx2-*eyfp* cells formed subpopulations (Fig. 3E and Fig. S3A and C). At 250 μM IPTG, 19.5% of the cells showed a high average fluorescence signal of 2.63 × 10^4^, whereas 78.5% of the cells had only a weak average fluorescence signal of 7.80 × 10^3^, and 2% of the cells possessed only background fluorescence. The overall averaged fluorescence signal of MB001/pEKEx2-*eyfp* cells including all subpopulations was 1.13 × 10^4^.

In the case of strain MB001/pJC1-P*_tac_*-*eyfp* (Fig. 3C), which was only analysed after cultivation without and with 250 μM IPTG, more than 99% of the cells cultivated in the presence of 250 μM IPTG showed high fluorescence, with an average signal intensity of 7.38 × 10^3^ and formed a homogenous population. But also in the absence of IPTG, about 90% of the cells showed a low fluorescence signal of 4.4 × 10^2^ above background, confirming the results of the Western blot shown in Fig. 2B. As pEKEx2-*eyfp* and pJC1-P*_tac_*-*eyfp* possess identical target gene expression determinants, the differences observed by flow cytometry presumably result from the different replicons. The vector pEKEx2 (Eikmanns *et al*., 1991) contains the replicon from plasmid pBL1 (Santamaria *et al*., 1984), whereas pJC1 (Cremer *et al*., 1990) contains the replicon from plasmid pHM1519 (Miwa *et al*., 1984), which presumably is identical with the one from plasmids pCG1 (Ozaki *et al*., 1984), pSR1 (Yoshihama *et al*., 1985) and pCG100 (Trautwetter and Blanco, 1991), as described previously (Nešvera and Pátek, 2008). Both replicons mediate replication in the rolling circle mode, but the pBL1 replicons belong to pIJ101/pJV1 family, whereas the pHM1519 replicon belongs to pNG2 family (Pátek and Nešvera, 2013). The copy number of pBL1 and similar plasmids was estimated to be between 10 and 30 copies per chromosome (Miwa *et al*., 1984; Santamaria *et al*., 1984), and that of pCG100 was also reported to be about 30 copies per chromosome (Trautwetter and Blanco, 1991). Apparently, the pNM1519 replicon is more stable than the pBL1 replicon, at least in the case of the expression vectors used in our study.

In the case of strain MB001(DE3)/pMKEx2-*eyfp* (Fig. 3D), less than 1% of the cells cultivated in the absence of IPTG showed fluorescence slightly above background. The fluorescence of cells cultivated in the presence of 5, 10, 15, 25, 50, 100 and 250 μM IPTG is shown in Fig. S3B and D. In the presence of 250 IPTG, 99.5% of the cells formed a very homogenous population, with an average fluorescence signal of about 5.22 × 10^4^. Compared with *C. glutamicum* MB001/pEKEx2-*eyfp* and MB001/pJC1-P*_tac_*-*eyfp*, the T7-based system showed a 4.6-fold and 7.1-fold higher eYFP signal after induction with 250 μM IPTG, respectively, whereas the signal in the uninduced state of the cells was at least 1.8-fold lower. These results confirm that the *C. glutamicum* T7 expression system allows tight repression of target gene expression in the absence of inducer, and a very uniform and strong expression level in the presence of inducer.

### Comparison of T7 RNAP-dependent expression of *eyfp* in *C. glutamicum* and *E. coli*

**Figure 4:**
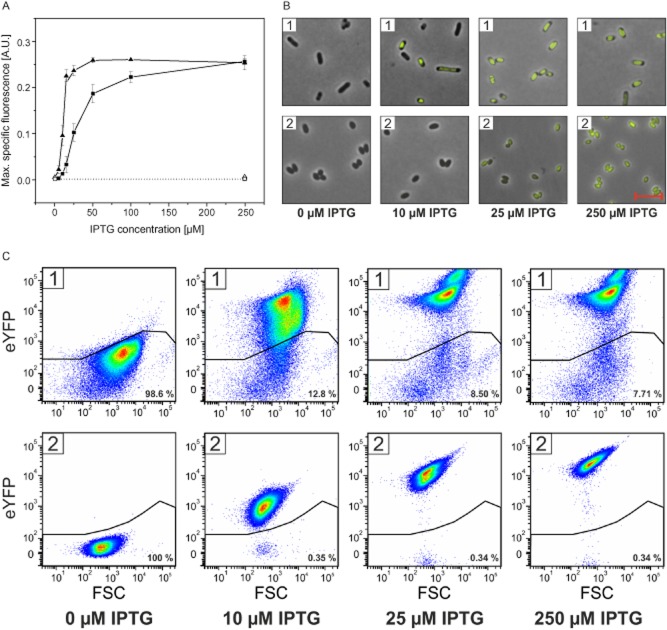
T7 RNAP-dependent expression of *eyfp* in *C. glutamicum* and *E. coli*. The strains *C. glutamicum* MB001/pMKEx2-*eyfp* (□), *C. glutamicum* MB001(DE3)/pMKEx2-*eyfp* (▪), *E. coli* BL21(DE3)/pMKEx2 (Δ) and *E. coli* BL21(DE3)/pMKEx2-*eyfp* (▴) were cultivated for 24 h aerobically in 2xTY medium using a BioLector system at 1200 r.p.m. and either 30°C (*C. glutamicum*) or 37°C (*E. coli*). Gene expression was induced 2 h after starting the cultivation by addition of 0–250 μM IPTG.A. After 24 h, the maximal specific eYFP fluorescence was determined (ratio of fluorescence emission at 532 nm and backscatter value at 620 nm). Mean values and standard deviations of at least three independent replicates are shown.B. Fluorescence microscopy images of *E. coli* BL21(DE3)/pMKEx2-*eyfp* (1) and *C. glutamicum* MB001(DE3)/pMKEx2-*eyfp* (2) cultivated with different IPTG concentrations. Images were taken with an exposure time of 40 ms. The red bar represents a length of 5 μm.C. Flow cytometry analysis of *E. coli* BL21(DE3)/pMKEx2-*eyfp* (1) and *C. glutamicum* MB001(DE3)/pMKEx2-*eyfp* (2) cultivated with different IPTG concentrations. Pseudo-coloured dot plots of eYFP fluorescence versus forward scatter are shown.

To compare the T7 RNAP-dependent expression system of *C. glutamicum* MB001(DE3) with the well-established *E. coli* BL21(DE3) system, the production of eYFP was analysed in both strains transformed with pMKEx2-*eyfp* and cultivated in 2xTY medium supplemented with different IPTG concentrations using the BioLector system (Fig. 4). The specific fluorescence of the culture in the absence of IPTG was lower for *C. glutamicum* MB001(DE3)/pMKEx2-*eyfp* (< 0.001) than for *E. coli* BL21(DE3)/pMKEx2-*eyfp* (0.003). The negative controls *C. glutamicum* MB001/pMKEx2-*eyfp* and *E. coli* BL21(DE3)/pMKEx2 showed only background fluorescence independent of the absence and presence of 250 μM IPTG (Fig. 4A). For *C. glutamicum* MB001(DE3)/pMKEx2-*eyfp* and *E. coli* BL21(DE3)/pMKEx2-*eyfp*, comparable maximal values of 0.26 ± 0.005 and 0.25 ± 0.002 were recorded for the specific eYFP fluorescence, but at different IPTG concentrations of 250 μM and 50 μM, respectively (Fig. 4A). Half-maximal specific fluorescence was obtained with 31 μM IPTG for strain MB001(DE3)/pMKEx2-*eyfp* and with 11 μM IPTG for strain BL21(DE3)/pMKEx2-*eypf*. This difference is probably due to the presence of lactose permease in *E. coli*, which presumably is involved in IPTG uptake and allows *E. coli* to obtain higher intracellular IPTG concentrations than a strain lacking *lacY* (Fernandez-Castane *et al*., 2012). In contrast to *E. coli*, *C. glutamicum* is unable to grow on lactose, but is able to do so when harbouring the *E. coli lac* operon, including *lacY*, which is essential for lactose uptake (Brabetz *et al*., [Bibr b9],[Bibr b10]).

Fluorescence microscopy (Fig. 4B) revealed that in the case of strain *E. coli* BL21(DE3)/pMKEx2-*eyfp* and *C. glutamicum* MB001(DE3)/pMKEx2-*eyfp*, almost all cells were fluorescent when cultivated in the presence of 250 μM IPTG. Whereas fluorescence was equally distributed over the entire cell in the case of the *C. glutamicum* strain, the majority of poles appeared non-fluorescent in the case of *E. coli* strain. The images taken at 10 and 25 μM IPTG confirm the results shown in Fig. 4A that *E. coli* requires lower IPTG concentrations for maximal expression.

Fluorescence-activated cell sorting analysis of cells of *E. coli* BL21(DE3)/pMKEx2-*eyfp* and *C. glutamicum* MB001(DE3)/pMKEx2-*eyfp* cultivated with 0, 10, 25 and 250 μM IPTG are depicted in Figs 4C and S4. Of the *E. coli* cells, 92% cultivated with 250 μM IPTG revealed an increased fluorescence, with an average signal intensity of 5.2 × 10^4^. In the case of *C. glutamicum*, more than 99% of the cells cultivated with 250 IPTG showed an increased fluorescence, with an average signal intensity of 2.8 × 10^4^. In contrast to the *E. coli* cells, the *C. glutamicum* cells formed a much more homogeneous population. When calculating the average fluorescence intensity of all analysed cells, the value for *C. glutamicum* MB001(DE3)/pMKEx2-*eyfp* (2.8 × 10^4^) was 1.8 times lower than the one for *E. coli* BL21(DE3)/pMKEx2-*eyfp* (5.0 × 10^4^). The FACS analysis of the strains cultivated in the absence of IPTG confirmed a lower basal *eyfp* expression in the *C. glutamicum* strain compared with the *E. coli* strain. Of the *E. coli* BL21(DE3)/pMKEx2-*eyfp* population, 1.4% showed an eYFP fluorescence above background, but none of the *C. glutamicum* MB001(DE3)/pMKEx2-*eyfp* cells.

### T7 RNAP-dependent overproduction of pyruvate kinase in *C. glutamicum* and *E. coli*

As an alternative target protein for analysing the properties of the newly established T7 expression system for *C. glutamicum*, we tested pyruvate kinase of *C. glutamicum*, which catalyses the conversion of phosphoenolpyruvate (PEP) and ADP to pyruvate and ATP. *Corynebacterium glutamicum* possesses a single *pyk* gene for pyruvate kinase (Gubler *et al*., 1994), which was amplified by PCR from chromosomal DNA of *C. glutamicum* MB001 with the oligonucleotides Pyk-for and Pyk-rev and cloned into pMKEx2 using BamHI and BsrGI restriction sites. The resulting plasmid pMKEx2-*pyk* was transferred into *C. glutamicum* MB001(DE3) and *E. coli* BL21(DE3), and the overproduction of pyruvate kinase was analysed by measuring the specific activity in crude extract.

**Figure 5:**
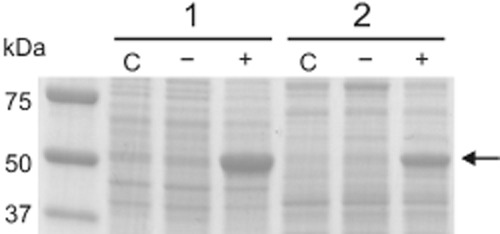
Coomassie-stained SDS-polyacrylamide gel for analysing overproduction of pyruvate kinase in *C. glutamicum* MB001(DE3)/pMKEx2-*pyk* (1) and *E. coli* BL21(DE3)/pMKEx2-*pyk* (2). The strains were cultivated in M9 medium with 2% (wt/vol) glucose (*E. coli*) or in CGXII medium with 4% (wt/vol) glucose (*C. glutamicum*). Strains labelled with ‘−’ were grown without IPTG, whereas strains labelled ‘+’ were supplemented with 250 μM IPTG when the cultures had reached an OD_600_ of 2. When the cultures had reached an OD_600_ of 5, cells were harvested and used for preparation of cell extracts. Ten microgram total protein of these extracts were subjected to SDS-PAGE. The samples labelled with ‘C’ represent control strains, either *C. glutamicum* MB001(DE3)/pMKEx2 (1) or *E. coli* BL21(DE3)/pMKEx2 (2), that were cultivated in the presence of 250 μM IPTG. The arrow indicates the predicted size for *C. glutamicum* pyruvate kinase (54.4 kDa).

The results presented in Table 3 show that the expression of the *pyk* gene on plasmid pMKEx2-*pyk* in the absence of IPTG led to a small increase of the endogenous pyruvate kinase activity of 0.5 U mg^−1^ in the case of *C. glutamicum* MB001(DE3) and of 0.9 U mg^−1^ in the case of *E. coli* BL21(DE3). When cultivated in the presence of 250 μM IPTG, the pyruvate kinase activity increased more than 40-fold to 135 U mg^−1^ in *C. glutamicum* carrying pMKEx2*-pyk* and 14-fold in *E. coli* carrying pMKEx2-*pyk*. In agreement with these data, SDS-PAGE of the cell extracts revealed a higher pyruvate kinase protein level in *C. glutamicum* compared with *E. coli* (Fig. 5). The more efficient overproduction of pyruvate kinase in the homologous host compared with *E. coli* might be due to a more efficient translation caused by differences in codon usage between the two species.

**Table 3:** Pyruvate kinase activity of different overexpression strains

Strain	Plasmid	Pyruvate kinase activity (U/mg) – IPTG	Increase (x-fold)^a^	Pyruvate kinase activity (U/mg) + 250 μM IPTG	Increase (x-fold)^b^
*C. glutamicum* MB001(DE3)	pMKEx2	n.d.	n.a.	2.6 ± 0.7	n.a.
*C. glutamicum* MB001(DE3)	pMKEx2-*pyk*	3.1 ± 0.2	1.2	135 ± 14	43.6
*E. coli* BL21(DE3)	pMKEx2	n.d.	n.a.	0.6 ± 0.1	n.a.
*E. coli* BL21(DE3)	pMKEx2-*pyk*	1.5 ± 0.2	2.8	20.9 ± 7.1	13.9

Specific activity was measured in crude extracts of cells harvested 4 h after addition or non-addition of IPTG. Mean values and standard deviations of at least three independent measurements are shown. n.d., not determined; n.a., not applicable.

a pMKEx2-*pyk* –IPTG versus pMKEx2 +IPTG.

a pMKEx2-*pyk* –IPTG versus +IPTG.

## Concluding remarks

In this study, a T7 RNAP-based expression system was developed for *C. glutamicum*. It is based on strain MB001(DE3), in which gene *1* encoding T7 RNAP is chromosomally encoded under control of the *lac*UV5 promoter, and the expression vector pMKEx2 carrying the T7*lac* promoter. Thus, both gene *1* and the target gene are repressed by LacI. Using *eyfp* as target gene, the new system allowed tightly IPTG-regulatable gene expression to levels that were about four times higher than those obtained with expression vectors using the *tac* promoter and the endogenous RNA polymerase. It thus probably represents the strongest overexpression system currently available for *C. glutamicum*. A particular feature of the new system was revealed by flow cytometry: IPTG induction led to the formation of very homogeneous populations, in which about 99% of the cells showed high expression of the target protein. Half-maximal induction was obtained with IPTG concentrations between 20 and 30 μM, depending on the medium used. The T7 RNAP-based system can be useful for the overproduction of proteins for subsequent purification, but also for metabolic engineering studies in which strong overproduction of certain genes is required.

## Experimental procedures

### Bacterial strains, plasmids and growth conditions

All bacterial strains and plasmids used in this study are listed in Table 1. *Escherichia coli* was grown at 37°C in a complex tryptone-yeast extract medium (2xTY) or in M9 minimal medium with 4% (wt/vol) glucose (Sambrook and Russell, 2001). *Corynebacterium glutamicum* was routinely cultivated at 30°C in 2xTY medium or in defined CGXII minimal medium with 4% (wt/vol) glucose (Keilhauer *et al*., 1993). If necessary, the media were supplemented with 50 mg l^−1^ kanamycin for *E. coli* and 25 mg l^−1^ kanamycin for *C. glutamicum*. *Escherichia coli* DH5α was used for plasmid construction, *E. coli* BL21(DE3) and *C. glutamicum* MB001(DE3) for overproduction of recombinant proteins. Cultures were inoculated to an optical density at 600 nm (OD_600_) of 1, and target gene induction was triggered by adding 0–250 μM IPTG to the culture at an OD_600_ of 2. To analyse *eyfp* expression, cells were grown as 800 μl cultures in 48-well microtitre plates (Flowerplates, m2p-labs, Baesweiler, Germany) at 80% humidity and 1200 r.p.m. using a BioLector system (m2p-labs, Baesweiler, Germany), which allows isochronal measurement of cell growth as backscattering light intensity at a wavelength of 620 nm and of eYFP fluorescence (ex/em 510/532 nm). For the overproduction of pyruvate kinase, all strains were cultivated at 120 r.p.m. and the required temperature in baffled 500 ml shake flasks with 50 ml CGXII or M9 medium, respectively.

### Recombinant DNA techniques

Standard DNA and cloning techniques were performed as described (Sambrook and Russell, 2001). Oligonucleotides were purchased from Eurofins MWG Operon (Ebersberg, Germany). Restriction enzymes (New England Biolabs, Frankfurt, Germany), shrimp alkaline phosphatase and Klenow fragment (both Thermo Scientific, Schwerte, Germany) were used according to the recommendations of the supplier. Introduction of a single point mutation in a pJC1 derivative was performed with the QuikChange Lightning Kit (Agilent Technologies, Waldbronn, Germany). Plasmid DNA of *E. coli* was isolated with QIAprep Spin Miniprep Kit (Qiagen, Hilden, Germany). Plasmid isolation from *C. glutamicum* was carried out with the same kit, but cells were pre-incubated in buffer P1 supplemented with 15 mg ml^−1^ (wt/vol) lysozyme for 2 h at 30°C. Purification of DNA fragments from agarose gels was done using the QIAex gel elution kit (Qiagen). RbCl-competent *E. coli* cells were transformed with plasmid DNA by the heat-shock method of Hanahan (1983), and *C. glutamicum* cells were transformed with plasmid DNA by electroporation as described previously (Tauch *et al*., 2002).

For the construction of plasmid pJC1-P*_tac_*-*eyfp*, the plasmid pJC1-venus-term (Baumgart *et al*., 2013a) was cut by NdeI and SalI to obtain a 6.58 kb fragment containing the same backbone (kanamycin resistance cassette, pCG1 replicon for *C. glutamicum*, pACYC177 replicon for *E. coli*) as pMKEx2. The expression cassette from plasmid pEKEx2-*eyfp* was amplified with oligonucleotides pEKEx-for and pEKEx-rev to obtain a 2.31 kb fragment containing the target gene *eyfp* under transcriptional control of the *tac* promoter and the *lacI* gene. After digestion with NdeI and SalI, this fragment was ligated with the 6.58 kb fragment from pJC1-venus-term to obtain pJC1-P*_tac_*-*eyfp*.

### Protein analysis

Recombinant protein production was analysed by SDS-PAGE. Cells were harvested by centrifugation and washed twice with lysis buffer (10 mM Tris-HCl, pH 8.0, 25 mM MgCl_2_, 200 mM NaCl). Afterwards, *C. glutamicum* and *E*. *coli* cells were disintegrated by beat beating using a Precellys 24 device (Peqlab Biotechnologie, Erlangen, Germany). Intact cells and cell debris were sedimented by centrifugation (13 000 *g*, 20 min), and the supernatant was used further. The concentration of intracellular proteins was determined with the BCA assay (BC Assay Protein Quantitation Kit, Uptima, Interchim, Montlucon, France) as described (Smith *et al*., 1985). Electrophoretic separation of proteins on SDS-polyacrylamide gels was performed by a standard procedure (Laemmli, 1970), and the gels were stained with Coomassie Brilliant Blue G-250 dye or used further for Western blot analysis. Immunological detection of eYFP was performed by using a polyclonal anti-GFP antibody (ab290, Abcam, Cambridge, UK) and a Cy5-conjugated goat-anti-rabbit antibody (GE Healthcare). Visualization and recording of fluorescent bands were performed using a Typhoon scanner (GE Healthcare) and the programme ImageQuant TL 7.0.

### Fluorescence microscopy

For fluorescence microscopy, cells were fixed on soft-agarose coated glass slides. Images were taken on a Zeiss Axioplan 2 imaging microscope that was equipped with an AxioCam MRm camera and a Plan-Apochromat 100×, 1.40 Oil Ph3 immersion objective. Digital images were acquired and analysed with the AxioVision 4.6 software (Zeiss, Göttingen, Germany).

### Flow cytometry

Cells were grown under appropriate conditions in a BioLector system, harvested after 24 h and diluted to an OD_600_ below 0.1 with sterile phosphate-buffered saline (37 mM NaCl, 2.7 mM KaCl, 10 mM Na_2_HPO_4_, 1.8 mM KH_2_PO_4_, pH 7.4). Expression of *eyfp* was analysed using a FACS ARIA II high-speed cell sorter (BD Biosciences, Franklin Lakes, NJ, USA) and the BD diva 6.1.3 software by measuring the eYFP fluorescence of single cells with an excitation wavelength of 488 nm and an emission wavelength of 533 ± 15 nm at a sample pressure of 70 psi. A threshold was set to exclude non-bacterial particles on the basis of forward versus side scatter area. There were 100 000 cells analysed for each measurement with a flow rate of 2000–4000 cells/s.

### Pyruvate kinase assay

Pyruvate kinase activity was determined spectrophotometrically using a coupled enzymatic assay with l-lactate dehydrogenase. The rate of NADH consumption was measured using an Infinite 200 PRO reader (Tecan, Männedorf, Switzerland) as the decrease of NADH absorbance at 340 nm (ε_NADH_ = 6.22 mM^−1^ cm^−1^). The assay mixture contained 100 mM Tris-HCl buffer (pH 7.3), 15 mM MgCl_2_, 1 mM ADP, 0.4 mM NADH, 5 U l-lactate dehydrogenase from pig heart, and 10 or 20 μl of cell extract (corresponding to 0.1–0.2 mg protein) in a total volume of 150 μl. The reaction was started by addition of 12 mM PEP. One unit of pyruvate kinase activity is defined as the amount of enzyme that converted 1 μmol of PEP to pyruvate per minute.

## Supporting Information

**Fig. S1.** Sequence of the 4,46 kb fragment amplified from the genome of *E. coli* BL21(DE3) and inserted into the intergenic region of cg1122-cg1121 of *C. glutamicum* MB001. 70 bp and 85 bp of the flanking *C. glutamicum* genome region are also shown with a grey background. The XhoI and EcoRI restriction sites used for cloning are shown in bold. **Fig. S2.** Growth of *C. glutamicum* MB001/pEKEx2-*eyfp* (A) and *C. glutamicum* MB001(DE3)/pMKEx2-*eyfp* (B). The strains were inoculated to an OD600 of 1 and cultivated for 24 h at 30°C in CGXII minimal medium with 4% (wt/vol) glucose using a BioLector system. Induction of *eyfp* expression was triggered by adding 0 μM (▪), 50 μM (), 100 μM •(▴), or 250 μM (◊) IPTG to the cultures after 2 h. **Fig. S3.** Analysis of heterologous eYFP production in the *C. glutamicum* strains MB001/pEKEx2-*eyfp* (A) and MB001(DE3)/pMKEx2-*eyfp* (B) at the single-cell level by flow cytometry. The strains were cultivated for 24 h at 30°C in CGXII minimal medium with 4% (wt/vol) glucose using a BioLector system. Induction of *eyfp* expression was triggered by adding the indicated concentrations of IPTG to the cultures after 2 h. Dot blots from FACS analysis (excitation at 488 nm, emission at 533 nm) of at least 100 000 cells of each strain displaying the eYFP fluorescence signal against the forward scatter signal (FSC). The gate used to define non-fluorescent cells was set with *C. glutamicum* MB001(DE3)/pMKEx2 with 100% of the cells falling into this gate (data not shown). The number inside the panels indicates the percentage of non-fluorescent cells inside this gate. In panels C and D, histograms of strains MB001/pEKEx2-*eyfp* (C) and MB001(DE3)/pMKEx2-*eyfp* (D) cultivated without IPTG or in the presence of 10, 25 and 250 μM IPTG are shown. The orange peaks indicate the background fluorescence set with strain MB001(DE3)/pMKEx2. The number of cells is plotted versus eYFP fluorescence. **Fig. S4.** Analysis of heterologous eYFP production in *C. glutamicum* MB001(DE3)/pMKEx2-*eyfp* (A) and *E. coli* BL21(DE3)/pEKEx2-*eyfp* (B) at the single-cell level. The strains were cultivated for 24 h at 30°C in 2xTY medium using a BioLector system. Induction of *eyfp* expression was triggered by adding the indicated concentrations of IPTG to the cultures after 2 h. Dot plots from FACS analysis (excitation at 488 nm, emission at 533 nm) of at least 100 000 cells of each strain displaying the eYFP signal against the forward scatter signal (FSC) are shown. The gate used to define non-fluorescent cells was set with *C. glutamicum* MB001/pMKEx2 or *E. coli* BL21(DE3)/pMKEx2, respectively, with 100% of the cells falling into this gate (data not shown). The number inside the panels indicates the percentage of non-fluorescent cells. In panels C and D, histograms of strains *E. coli* BL21(DE3)/pMKEx2-*eyfp* (C) and *C. glutamicum* MB001(DE3)/pMKEx2-*eyfp* (D) cultivated without IPTG or in the presence of 10, 25 and 250 μM IPTG are shown. The orange peaks indicate the background fluorescence set with strain *E. coli* BL21(DE3)/pMKEx2 and *C. glutamicum* MB001(DE3)/pMKEx2. The number of cells is plotted versus eYFP fluorescence.
