# Supplementary material for: A chromosomally encoded T7 RNA polymerase-dependent gene expression system for Corynebacterium glutamicum: construction and comparative evaluation at the single-cell level
Source: Microb Biotechnol. 2014 Dec 9;8(2):253–65. doi: 10.1111/1751-7915.12236 (PMC4353339; doi:10.1111/1751-7915.12236)
Supplement: Supplementary file 1 [file mbt20008-0253-sd1.pdf]

Supplemental material to Kortmann et al. 2014

cg1122 <--

GGAAATAGGGGCCTTTTGTTGTCTTCTCCTGGAGGCTATTTAAGAAGTTTAAATTGTGTCCATGAGTTCG
CTCGAGAACTGCGCAACTCGTGAAAGGTAGGCGGATCCAGATCCCGGACACCATCGAATGGCGCAAAACC
TTTCGCGGTATGGCATGATAGCGCCCGGAAGAGAGTCAATTCAGGGTGGTGAATGTGAAACCAGTAACGT
TATACGATGTCGCAGAGTATGCCGGTGTCTCTTATCAGACCGTTTCCCGCGTGGTGAACCAGGCCAGCCA
CGTTTCTGCGAAAACGCGGGAAAAAGTGGAAGCGGCGATGGCGGAGCTGAATTACATTCCCAACCGCGTG
GCACAACAACTGGCGGGCAAACAGTCGTTGCTGATTGGCGTTGCCACCTCCAGTCTGGCCCTGCACGCGC
CGTCGCAAATTGTCGCGGCGATTAAATCTCGCGCCGATCAACTGGGTGCCAGCGTGGTGGTGTCGATGGT
AGAACGAAGCGGCGTCGAAGCCTGTAAAGCGGCGGTGCACAATCTTCTCGCGCAACGCGTCAGTGGGCTG
ATCATTAACTATCCGCTGGATGACCAGGATGCCATTGCTGTGGAAGCTGCCTGCACTAATGTTCCGGCGT
TATTTCTTGATGTCTCTGACCAGACACCCATCAACAGTATTATTTTCTCCCATGAAGACGGTACGCGACT
GGGCGTGGAGCATCTGGTCGCATTGGGTCACCAGCAAATCGCGCTGTTAGCGGGCCCATTAAGTTCTGTC
TCGGCGCGTCTGCGTCTGGCTGGCTGGCATAAATATCTCACTCGCAATCAAATTCAGCCGATAGCGGAAC
GGGAAGGCGACTGGAGTGCCATGTCCGGTTTTCAACAAACCATGCAAATGCTGAATGAGGGCATCGTTCC
CACTGCGATGCTGGTTGCCAACGATCAGATGGCGCTGGGCGCAATGCGCGCCATTACCGAGTCCGGGCTG
CGCGTTGGTGCGGATATCTCGGTAGTGGGATACGACGATACCGAAGACAGCTCATGTTATATCCCGCCGT
TAACCACCATCAAACAGGATTTTCGCCTGCTGGGGCAAACCAGCGTGGACCGCTTGCTGCAACTCTCTCA
GGGCCAGGCGGTGAAGGGCAATCAGCTGTTGCCCGTCTCACTGGTGAAAAGAAAAACCACCCTGGCGCCC
AATACGCAAACCGCCTCTCCCCGCGCGTTGGCCGATTCATTAATGCAGCTGGCACGACAGGTTTCCCGAC
TGGAAAGCGGGCAGTGAGCGCAACGCAATTAATGTAAGTTAGCTCACTCATTAGGCACCCCAGGCTTTAC
ACTTTATGCTTCCGGCTCGTATAATGTGTGGAATTGTGAGCGGATAACAATTTCACACAGGAAACAGCTA
TGACCATGATTACGGATTCACTGGCCGTCGTTTTACAACGTCGTGACTGGGAAAACCCTGGCGTTACCCA
ACTTAATCGCCTTGCAGCACATCCCCCTTTCGCCAGCTGGCGTAATAGCGAAGAGGCCCGCACCGATCGC
CCTTCCCAACAGTTGCGCAGCCTGAATGGCGAATGGCGCTTTGCCTGGTTTCCGGCACCAGAAGCGGTGC
CGGAAAGCTGGCTGGAGTGCGATCTTCCTGAGGCCGATACTGTCGTCGTCCCCTCAAACTGGCAGATGCA
CGGTTACGATGCGCCCATCTACACCAACGTGACCTATCCCATTACGGTCAATCCGCCGTTTGTTCCCACG
GAGAATCCGACGGGTTGTTACTCGCTCACATTTAATGTTGATGAAAGCTGGCTACAGGAAGGCCAGACGC
GAATTATTTTTGATGGCGTCGGGATCTGATCCGGATTTACTAACTGGAAGAGGCACTAAATGAACACGAT
TAACATCGCTAAGAACGACTTCTCTGACATCGAACTGGCTGCTATCCCGTTCAACACTCTGGCTGACCAT
TACGGTGAGCGTTTAGCTCGCGAACAGTTGGCCCTTGAGCATGAGTCTTACGAGATGGGTGAAGCACGCT
TCCGCAAGATGTTTGAGCGTCAACTTAAAGCTGGTGAGGTTGCGGATAACGCTGCCGCCAAGCCTCTCAT
CACTACCCTACTCCCTAAGATGATTGCACGCATCAACGACTGGTTTGAGGAAGTGAAAGCTAAGCGCGGC
AAGCGCCCGACAGCCTTCCAGTTCCTGCAAGAAATCAAGCCGGAAGCCGTAGCGTACATCACCATTAAGA
CCACTCTGGCTTGCCTAACCAGTGCTGACAATACAACCGTTCAGGCTGTAGCAAGCGCAATCGGTCGGGC
CATTGAGGACGAGGCTCGCTTCGGTCGTATCCGTGACCTTGAAGCTAAGCACTTCAAGAAAAACGTTGAG
GAACAACTCAACAAGCGCGTAGGGCACGTCTACAAGAAAGCATTTATGCAAGTTGTCGAGGCTGACATGC
TCTCTAAGGGTCTACTCGGTGGCGAGGCGTGGTCTTCGTGGCATAAGGAAGACTCTATTCATGTAGGAGT
ACGCTGCATCGAGATGCTCATTGAGTCAACCGGAATGGTTAGCTTACACCGCCAAAATGCTGGCGTAGTA
GGTCAAGACTCTGAGACTATCGAACTCGCACCTGAATACGCTGAGGCTATCGCAACCCGTGCAGGTGCGC
TGGCTGGCATCTCTCCGATGTTCCAACCTTGCGTAGTTCCTCCTAAGCCGTGGACTGGCATTACTGGTGG
TGGCTATTGGGCTAACGGTCGTCGTCCTCTGGCGCTGGTGCGTACTCACAGTAAGAAAGCACTGATGCGC
TACGAAGACGTTTACATGCCTGAGGTGTACAAAGCGATTAACATTGCGCAAAACACCGCATGGAAAATCA
ACAAGAAAGTCCTAGCGGTCGCCAACGTAATCACCAAGTGGAAGCATTGTCCGGTCGAGGACATCCCTGC
GATTGAGCGTGAAGAACTCCCGATGAAACCGGAAGACATCGACATGAATCCTGAGGCTCTCACCGCGTGG
AAACGTGCTGCCGCTGCTGTGTACCGCAAGGACAAGGCTCGCAAGTCTCGCCGTATCAGCCTTGAGTTCA
TGCTTGAGCAAGCCAATAAGTTTGCTAACCATAAGGCCATCTGGTTCCCTTACAACATGGACTGGCGCGG
TCGTGTTTACGCTGTGTCAATGTTCAACCCGCAAGGTAACGATATGACCAAAGGACTGCTTACGCTGGCG
AAAGGTAAACCAATCGGTAAGGAAGGTTACTACTGGCTGAAAATCCACGGTGCAAACTGTGCGGGTGTCG
ATAAGGTTCCGTTCCCTGAGCGCATCAAGTTCATTGAGGAAAACCACGAGAACATCATGGCTTGCGCTAA
GTCTCCACTGGAGAACACTTGGTGGGCTGAGCAAGATTCTCCGTTCTGCTTCCTTGCGTTCTGCTTTGAG

```
TACGCTGGGGTACAGCACCACGGCCTGAGCTATAACTGCTCCCTTCCGCTGGCGTTTGACGGGTCTTGCT
CTGGCATCCAGCACTTCTCCGCGATGCTCCGAGATGAGGTAGGTGGTCGCGCGGTTAACTTGCTTCCTAG
TGAAACCGTTCAGGACATCTACGGGATTGTTGCTAAGAAAGTCAACGAGATTCTACAAGCAGACGCAATC
AATGGGACCGATAACGAAGTAGTTACCGTGACCGATGAGAACACTGGTGAAATCTCTGAGAAAGTCAAGC
TGGGCACTAAGGCACTGGCTGGTCAATGGCTGGCTTACGGTGTTACTCGCAGTGTGACTAAGCGTTCAGT
CATGACGCTGGCTTACGGGTCCAAAGAGTTCGGCTTCCGTCAACAAGTGCTGGAAGATACCATTCAGCCA
GCTATTGATTCCGGCAAGGGTCTGATGTTCACTCAGCCGAATCAGGCTGCTGGATACATGGCTAAGCTGA
TTTGGGAATCTGTGAGCGTGACGGTGGTAGCTGCGGTTGAAGCAATGAACTGGCTTAAGTCTGCTGCTAA
GCTGCTGGCTGCTGAGGTCAAAGATAAGAAGACTGGAGAGATTCTTCGCAAGCGTTGCGCTGTGCATTGG
GTAACTCCTGATGGTTTCCCTGTGTGGCAGGAATACAAGAAGCCTATTCAGACGCGCTTGAACCTGATGT
TCCTCGGTCAGTTCCGCTTACAGCCTACCATTAACACCAACAAAGATAGCGAGATTGATGCACACAAACA
GGAGTCTGGTATCGCTCCTAACTTTGTACACAGCCAAGACGGTAGCCACCTTCGTAAGACTGTAGTGTGG
GCACACGAGAAGTACGGAATCGAATCTTTTGCACTGATTCACGACTCCTTCGGTACCATTCCGGCTGACG
CTGCGAACCTGTTCAAAGCAGTGCGCGAAACTATGGTTGACACATATGAGTCTTGTGATGTACTGGCTGA
TTTCTACGACCAGTTCGCTGACCAGTTGCACGAGTCTCAATTGGACAAAATGCCAGCACTTCCGGCTAAA
GGTAACTTGAACCTCCGTGACATCTTAGAGTCGGACTTCGCGTTCGCGTAACGAATTCGCGTATGGCAAT
GACAGTTTGAGACGGCCACAGGCGATTCTGAGAAGCCATTTTCTTTGGGCGCCGTGGCAGTTTTTATTGG
--> cg1121
```

Fig. S1. Sequence of the 4,46 kb fragment amplified from the genome of *E. coli* BL21(DE3) and inserted into the intergenic region of cg1122-cg1121 of *C. glutamicum* MB001. 70 bp and 85 bp of the flanking *C. glutamicum* genome region are also shown with a grey background. The XhoI and EcoRI restriction sites used for cloning are shown in bold.

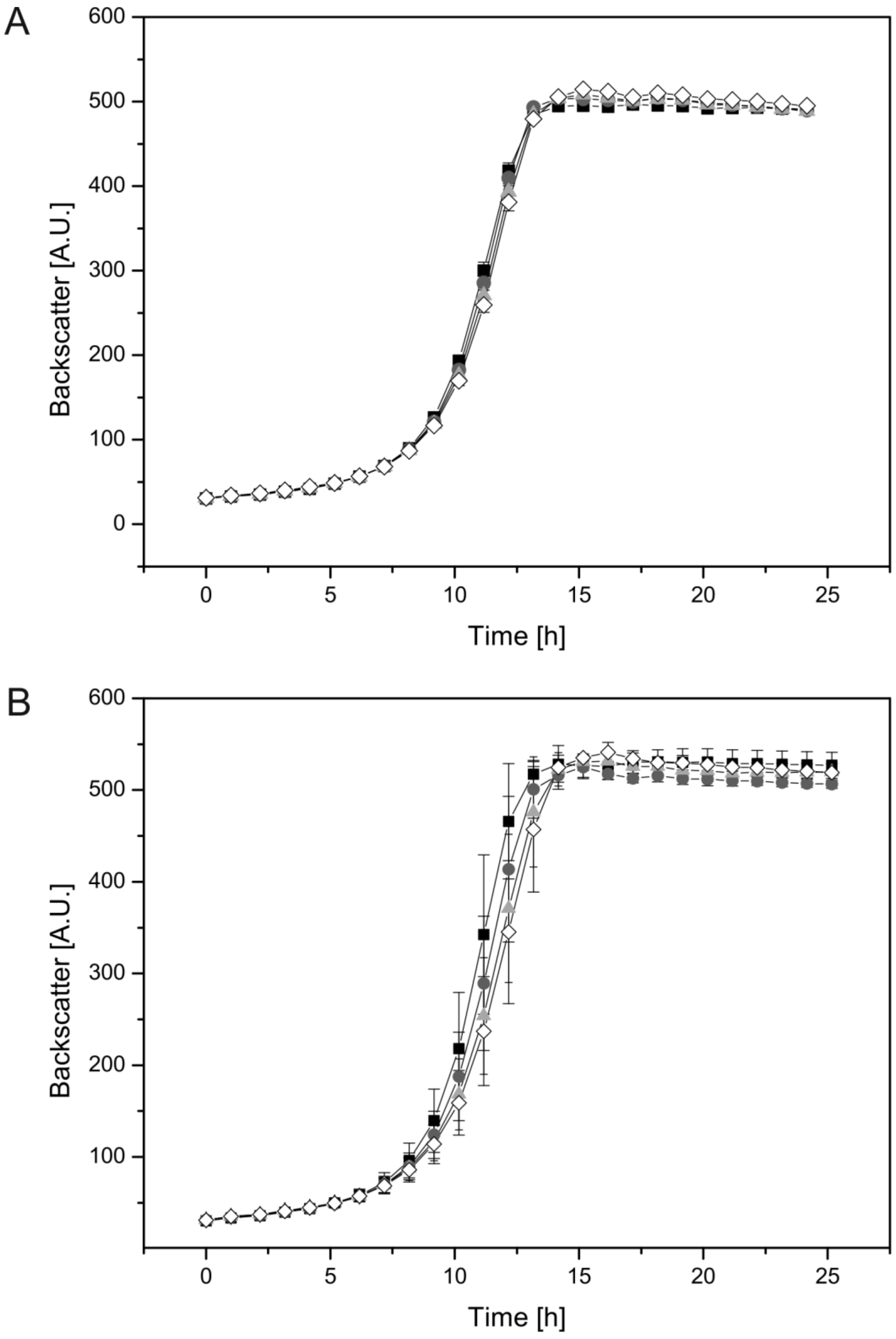

Fig. S2. Growth of *C. glutamicum* MB001/pEKEx2-*eyfp* (A) and *C. glutamicum* MB001(DE3)/pMKEx2-*eyfp* (B). The strains were inoculated to an OD_{600} of 1 and cultivated for 24 h at 30°C in CGXII minimal medium with 4% (wt/vol) glucose using a BioLector system. Induction of *eyfp* expression was triggered by adding 0 µM (■), 50 µM (●), 100 µM (▲), or 250 µM (◊) IPTG to the cultures after 2 h.

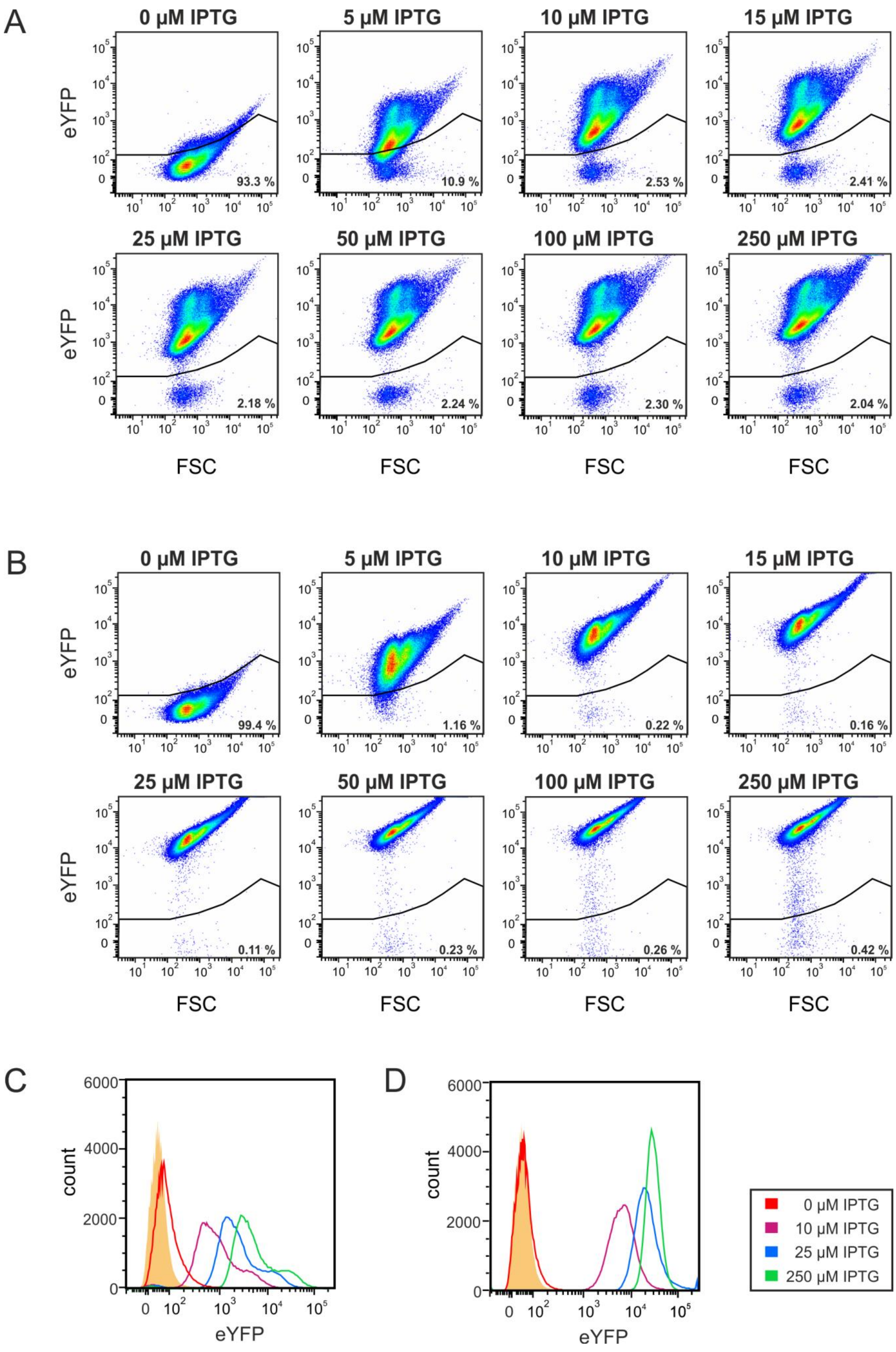
A
0 µM IPTG
5 µM IPTG
10 µM IPTG
15 µM IPTG
93.3 %
10.9 %
2.53 %
2.41 %
25 µM IPTG
50 µM IPTG
100 µM IPTG
250 µM IPTG
2.18 %
2.24 %
2.30 %
2.04 %
eYFP
FSC
B
0 µM IPTG
5 µM IPTG
10 µM IPTG
15 µM IPTG
99.4 %
1.16 %
0.22 %
0.16 %
25 µM IPTG
50 µM IPTG
100 µM IPTG
250 µM IPTG
0.11 %
0.23 %
0.26 %
0.42 %
eYFP
FSC
C
count
eYFP
D
count
eYFP
0 µM IPTG
10 µM IPTG
25 µM IPTG
250 µM IPTG

Fig. S3. Analysis of heterologous eYFP production in the *C. glutamicum* strains MB001/pEKEx2-*eyfp* (A) and MB001(DE3)/pMKEx2-*eyfp* (B) at the single cell level by flow cytometry. The strains were cultivated for 24 h at 30°C in CGXII minimal medium with 4% (wt/vol) glucose using a BioLector system. Induction of *eyfp* expression was triggered by adding the indicated concentrations of IPTG to the cultures after 2 h. Dot blots from FACS analysis (excitation at 488 nm, emission at 533 nm) of at least 100,000 cells of each strain displaying the eYFP fluorescence signal against the forward scatter signal (FSC). The gate used to define non-fluorescent cells was set with *C. glutamicum* MB001(DE3)/pMKEx2 with 100 % of the cells falling into this gate (data not shown). The number inside the panels indicates the percentage of non-fluorescent cells inside this gate. In panels C and D, histograms of strains MB001/pEKEx2-*eyfp* (C) and MB001(DE3)/pMKEx2-*eyfp* (D) cultivated without IPTG or in the presence of 10, 25, and 250 µM IPTG are shown. The orange peaks indicate the background fluorescence set with strain MB001(DE3)/pMKEx2. The number of cells is plotted vs. eYFP fluorescence.

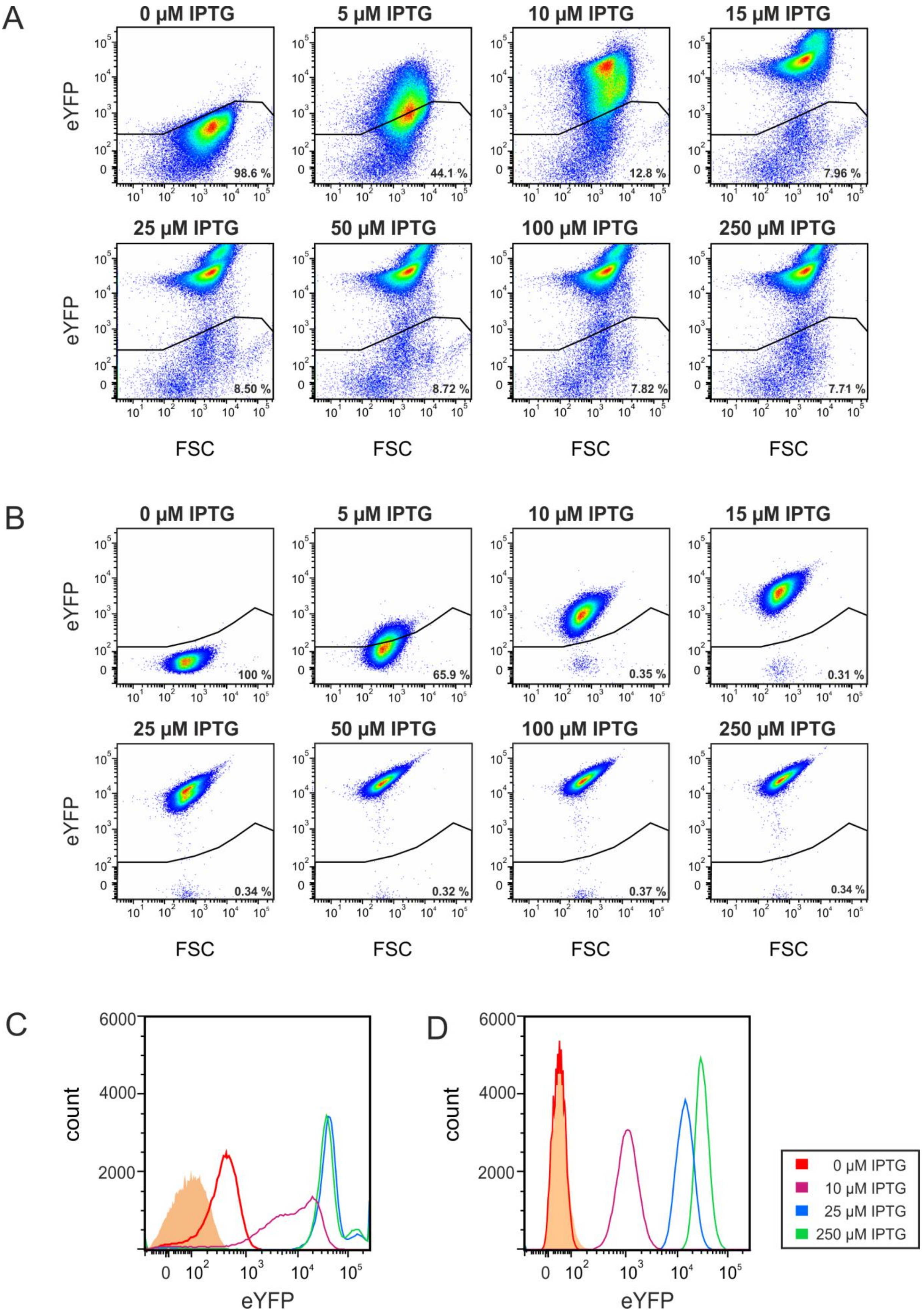
A
0 µM IPTG
5 µM IPTG
10 µM IPTG
15 µM IPTG
98.6 %
44.1 %
12.8 %
7.96 %
25 µM IPTG
50 µM IPTG
100 µM IPTG
250 µM IPTG
8.50 %
8.72 %
7.82 %
7.71 %
eYFP
FSC
B
0 µM IPTG
5 µM IPTG
10 µM IPTG
15 µM IPTG
100 %
65.9 %
0.35 %
0.31 %
25 µM IPTG
50 µM IPTG
100 µM IPTG
250 µM IPTG
0.34 %
0.32 %
0.37 %
0.34 %
eYFP
FSC
C
count
eYFP
D
count
eYFP
0 µM IPTG
10 µM IPTG
25 µM IPTG
250 µM IPTG

Fig. S4. Analysis of heterologous eYFP production in *C. glutamicum* MB001(DE3)/pMKEx2-*eyfp* (A) and *E. coli* BL21(DE3)/pEKEx2-*eyfp* (B) at the single cell level. The strains were cultivated for 24 h at 30°C in 2xTY medium using a BioLector system. Induction of *eyfp* expression was triggered by adding the indicated concentrations of IPTG to the cultures after 2 h. Dot plots from FACS analysis (excitation at 488 nm, emission at 533 nm) of at least 100,000 cells of each strain displaying the eYFP signal against the forward scatter signal (FSC) are shown. The gate used to define non-fluorescent cells was set with *C. glutamicum* MB001/pMKEx2 or *E. coli* BL21(DE3)/pMKEx2, respectively, with 100 % of the cells falling into this gate (data not shown). The number inside the panels indicates the percentage of non-fluorescent cells. In panels C and D, histograms of strains *E. coli* BL21(DE3)/pMKEx2-*eyfp* (C) and *C. glutamicum* MB001(DE3)/pMKEx2-*eyfp* (D) cultivated without IPTG or in the presence of 10, 25, and 250 µM IPTG are shown. The orange peaks indicate the background fluorescence set with strain strain *E. coli* BL21(DE3)/pMKEx2 and *C. glutamicum* MB001(DE3)/pMKEx2. The number of cells is plotted vs. eYFP fluorescence.
